# Voltage-Dependent Anion Selective Channel Isoforms in Yeast: Expression, Structure, and Functions

**DOI:** 10.3389/fphys.2021.675708

**Published:** 2021-05-19

**Authors:** Maria Carmela Di Rosa, Francesca Guarino, Stefano Conti Nibali, Andrea Magrì, Vito De Pinto

**Affiliations:** ^1^Department of Biomedical and Biotechnological Sciences, University of Catania, Catania, Italy; ^2^we.MitoBiotech S.R.L., Catania, Italy; ^3^Department of Biological, Geological and Environmental Sciences, University of Catania, Catania, Italy

**Keywords:** porin, VDAC, mitochondria, electrophyiology, yeast, outer mitochondrial membrane

## Abstract

Mitochondrial porins, also known as voltage-dependent anion selective channels (VDACs), are pore-forming molecules of the outer mitochondrial membranes, involved in the regulation of metabolic flux between cytosol and mitochondria. Playing such an essential role, VDAC proteins are evolutionary conserved and isoforms are present in numerous species. The quest for specific function(s) related to the raise of multiple isoforms is an intriguing theme. The yeast *Saccharomyces cerevisiae* genome is endowed with two different VDAC genes encoding for two distinct porin isoforms, definitely less characterized in comparison to mammalian counterpart. While yVDAC1 has been extensively studied, the second isoform, yVDAC2, is much less expressed, and has a still misunderstood function. This review will recapitulate the known and poorly known information in the literature, in the light of the growing interest about the features of VDAC isoforms in the cell.

## Introduction

The passive diffusion of small hydrophilic molecules throughout outer membranes (OM) of Gram-negative bacteria, mitochondria and chloroplast is provided by the presence of integral membrane proteins commonly named *porins*. Characterized by a cylindrical shape, porins were firstly discovered in prokaryotes (Nakae, 1976) and subsequently in mitochondria (Schein et al., 1976; Colombini, 1979) and chloroplast (Smack and Colombini, 1985), supporting the endosymbiotic theory. Porins are generally arranged in a conserved β-barrel structure, with polar amino acids facing the hydrophilic compartments counterbalanced by non-polar residues in the hydrophobic membrane core (Benz, 1989; Rosenbusch, 1990; Zeth and Thein, 2010).

The first mitochondrial porin was identified in the unicellular ciliate *Paramecium tetraurelia* by Schein et al. (1976). In artificial membranes, the protein showed a maximal conductance at the transmembrane potential close to zero, which decreased as a function of both positive and negative voltage applied (Schein et al., 1976). Furthermore, the channel exhibited a slight preference for anions over cations in the high-conducting state (Schein et al., 1976; Benz et al., 1988). Given these electrophysiological features, mitochondrial porin was then named Voltage-Dependent Anion selective Channel (VDAC).

VDACs are ubiquitously expressed proteins of about 28–32 kDa, with an estimated pore dimension of ∼3–3.5 nm in diameter and ∼4–4.5 nm in height. The number of VDAC isoforms varies significantly in many species, ranging from one or two in yeast, three in mammals and up to five in plants (Young et al., 2007). Anyway, they represent the most abundant protein family of the outer mitochondrial membrane (OMM), accounting for ∼50% of the total protein content (Mannella, 1998; Gonçalves et al., 2007). This confers the typical sieve-like aspect to the OMM, as revealed by atomic force microscopy experiments (Gonçalves et al., 2007, 2008).

While human and murine VDACs were extensively studied, the same was not for the *Saccharomyces cerevisiae* counterpart. *S. cerevisiae*, also known as the Baker’s yeast, is a unicellular organism widely employed as a eukaryotic model. Its genome was completely sequenced in 1996 (Goffeau et al., 1996), making the genetic manipulation simpler through recombination techniques. Furthermore, most of the metabolic and cellular pathways, especially those involving mitochondria biogenesis and function, are conserved. This has led to define yeast “a smaller but not lower eukaryote” (Rine, 1989).

In the lights of these considerations, in this review we summarized all the literature information available so far about the structure, the electrophysiological features and the peculiar functions of the two VDAC isoforms expressed by the yeast *S. cerevisiae*.

## The Structure and Functions of VDAC Proteins

From the time of their discovery, many hypotheses were formulated about VDAC three-dimensional structure. The alternation of hydrophobic and hydrophilic residues, as revealed by the sequence analysis, and a set of single-point mutagenesis experiments allowed the development of the first model, consisting in a transmembrane barrel made of 12 antiparallel β-strands and one amphipathic is α-helix (Blachly-Dyson et al., 1990; Thomas et al., 1993). In the early 2000s, by using computational approaches, *Neurospora crassa* and *S. cerevisiae* VDACs were modeled onto bacterial porins structures available at that time, predicting a 16 β-strands barrel structure with a globular α-helix corresponding to the first amino acid residues of the N-terminal domain (Casadio et al., 2002). The specific structure of N-terminus, already predicted by the first studies (Blachly-Dyson et al., 1990; Thomas et al., 1993), was experimentally confirmed by circular dichroism experiments performed by independent groups (Guo et al., 1995; Bay and Court, 2002; De Pinto et al., 2007). However, only several years later the three-dimensional structures of murine and human VDAC1 (Bayrhuber et al., 2008; Hiller et al., 2008; Ujwal et al., 2008) and zebrafish VDAC2 (Schredelseker et al., 2014) were determined by NMR spectroscopy, X-ray crystallography or a combination of these two techniques.

The β-barrel pore structure of VDAC proteins is built by 19 β-strands connected to each other by short turns and loops. This makes mitochondrial porins significantly different from bacterial general porins, which have an even, variable number of β-strands, commonly between 14 and 18 (Achouak et al., 2001; Nikaido, 2003). In VDAC, strands are anti-parallel except for the first and the last one, showing instead a parallel orientation. As predicted, in the first 26 N-terminal residues two short α-helix stretches were found. Although there are several differences in the specific location among the models, all authors agree that N-terminus does not take part in the barrel formation, as evinced also from the superposition of human and murine VDAC1 (Zeth and Zachariae, 2018). According to Bayrhuber et al. (2008) the sequence 7–17 (at the N-terminal end) is horizontally oriented inside the barrel and the sequence 3–7 contacts the pore wall. Similar findings have been found in the other models, where the presence of N-terminus within the channel lumen was justified by the presence of a specific hydrogen-bonding pattern between it and several specific residues located in different strands of the barrel (Ujwal et al., 2008) and/or by hydrophobic interactions (Hiller and Wagner, 2009).

As farther detailed, a putative role in the channel gating was assigned to the N-terminus (Shuvo et al., 2016). In fact, the domain is connected to the barrel by a glycine-rich motif, which confers flexibility. It is thus believed that N-terminal domain is capable to leave the lumen and to partially expose itself to the cytosolic environment, possibly mediating the interaction with the membrane or other proteins (Geula et al., 2012; Manzo et al., 2018). This hypothesis is supported by the transmission electron microscopy work by Guo et al. (1995) and, more recently, by the definition of VDAC topology within the OMM (Tomasello et al., 2013).

The pore allows the passive diffusion through the OMM of small ions (Na^+^, Cl^–^, and K^+^) and metabolites up to ∼5,000 Da, including ATP/ADP and nucleotides, intermediates of Krebs’ cycle (glutamate, pyruvate, succinate, malate) and NAD^+^/NADH (Benz, 1994; Hodge and Colombini, 1997; Rostovtseva and Colombini, 1997; Gincel and Shoshan-Barmatz, 2004). Other small molecules are instead capable to modulate the pore activity and/or interaction of VDACs with cytosolic proteins and enzymes. By binding the channel, cholesterol preserves the structural integrity of VDAC and facilitates its insertion in lipid bilayers (De Pinto et al., 1989a; Popp et al., 1995; Hiller et al., 2008). Being a component of the OMM, cholesterol amount may vary according to the conditions, affecting in turn VDAC functionality (Baggetto et al., 1992; Pastorino and Hoek, 2008).

In this perspective, VDACs are widely considered essential for the maintenance of the mitochondrial bioenergetic and the communication between the organelle and the rest of the cell (as reviewed in Shoshan-Barmatz et al., 2010; De Pinto, 2021).

## Voltage-Dependent Anion Selective Channels Have Isoform-Specific Functions

Both VDAC genes and proteins are evolutionary conserved. The three different mammalian VDAC isoforms are encoded by independent genes, each characterized by a similar intron-exon organization. VDAC2 gene has an additional pre-sequence placed upstream of the first exon that confers to the protein a 11-amino acids longer N-terminus (Messina et al., 2012). Furthermore, the proteins are characterized by high intra- and inter-species sequence conservation. For instance, mammalian isoforms show up to ∼75% of sequence similarity, while yeast and human VDAC1 share about 70% of similar sequence (Young et al., 2007; Messina et al., 2012). This implies that all VDAC proteins should have similar structure/functions, as arises from computational simulations made for all the proteins for which the three-dimensional structure is not available yet (De Pinto et al., 2010; Guardiani et al., 2018). Accordingly, VDACs from mice, yeast, fruit fly, and human can substitute for each other in the regulation of metabolic fluxes if expressed in yeast mitochondria, as demonstrated by complementation assays performed in porin-less strain(s) on non-fermentable carbon sources (i.e., glycerol) at the restrictive temperature of 37°C (Sampson et al., 1997; Xu et al., 1999; Reina et al., 2010; Leggio et al., 2018). At the same time, the simultaneous presence of different isoforms has raised the question of distinct and non-redundant functions for each VDAC. While this issue is unexplained for yeast, several hypotheses have been put forward for high eukaryotes.

In mammals, VDACs show a tissue-specific distribution in which VDAC1 is the most ubiquitous (Cesar and Wilson, 2004; De Pinto et al., 2010). While controlling the overall permeability of OMM (Tomasello et al., 2009) and participating in Ca^2+^ homeostasis (De Stefani et al., 2012), VDAC1 interacts with proteins of Bcl-2 family and hexokinases, playing a crucial role for the activation of apoptosis (Shimizu et al., 2001; Abu-Hamad et al., 2008; Huang et al., 2013). Upon specific stimuli, VDAC1 undergoes oligomerization allowing MOM permeabilization, as well as the release of cytochrome c and/or mitochondrial DNA fragments (Keinan et al., 2010;. Kim et al., 2019).

VDAC2 was initially indicated as a pro-survival protein, being able to prevent the activation of the pro-apoptotic protein Bak (Cheng et al., 2003). In the last years, however, a mechanism in which VDAC2 is necessary for the activation of Bax, another pro-apoptotic member of Bcl-2 family, was proposed (Ma et al., 2014; Chin et al., 2018).

On the contrary, the specific role of VDAC3 remains not completely clarified yet. This isoform has specific and peculiar features: for example, in non-reducing conditions it forms small pores of about 90 pS in artificial membranes (Checchetto et al., 2014) while, in yeast devoid of endogenous porins, it complements the growth defect only partially (Sampson et al., 1997; Reina et al., 2010). In the last years, our group carried out site-direct mutagenesis experiments on human VDAC3 aimed at replacing cysteine with alanine residues. Cysteines, indeed, can undergo oxidation/reduction according to the environment. Mutations of single or multiple cysteines significantly increased the conductance of VDAC3 up to similar or identical values to those shown by the other isoforms (Reina et al., 2016a; Queralt-Martín et al., 2020), suggesting that the pattern of post-translational modifications (PTMs) modulates VDAC3 activity (Okazaki et al., 2015; Reina et al., 2016a). This hypotesis was later confirmed by mass spectrometry (Saletti et al., 2017; Pittalà et al., 2020). Given also the specific interaction of VDAC3 with stress sensor and redox-mediating enzymes (Messina et al., 2014), this isoform was indicated as a putative mitochondrial sensor of the oxidative stress (De Pinto et al., 2016; Reina et al., 2016b, 2020).

The pattern of cysteine oxidation was never studied in yeast. Isoform 1 has only two cysteines similar to mammal VDAC1. The same holds for yVDAC2, whose PTMs study by mass spectrometry is practically hindered by its paucity in the usual yeast strains.

## The VDAC Genes and Proteins in Yeast

Differently from mammals, *S. cerevisiae* genome is endowed with two different genes encoding for two distinct porin isoforms. As summarized in Table 1, the so-called *POR* genes are located in different chromosomes and they are very similar in length. A comparative genomic analysis has suggested that isoforms have been originated from genome duplication during the evolution. This phenomenon was postulated for *S. cerevisiae* and *Candida glabrata* but not for other Saccharomycetales fungi such as *Kluyveromyces lactis* or *S. pombe* in which VDAC paralogs were not detected (Kellis et al., 2004). At the same time, in *C. glabrata* the gene encoding for the second VDAC isoform is highly degenerate, raising the specific question about the maintenance of both *POR* copies in *S. cerevisiae* (Young et al., 2007).

**TABLE 1:** Main features of POR genes and proteins.

**Gene**	**ORF**	**Location**	**Position**	**Size (bp)**	**Protein length (aa)**
*POR1*	YNL055C	chrXIV	517.994–518.845	852	283
*POR2*	YIL114C	chrIX	149.143–149.988	846	281

*Genetic information about POR genes and the coorrespondent proteins. Data were taken from Saccharomices Genome Database (https://www.yeastgenome.org).*

Being encoded by the nuclear genome, all VDACs are synthesized by cytosolic ribosomes and subsequently imported into the OMM by specific evolutionary conserved protein complexes (Ulrich and Rapaport, 2015). This process was in-depth studied using the yeast as a cellular model. Briefly, the translocase of outer membrane (TOM) complex recognizes VDAC precursors onto OMM surface and drives the protein translocation through the main TOM complex subunit, Tom40 (Pfanner et al., 2004; Chacinska et al., 2009), which is itself a β-barrel protein (Araiso et al., 2019; Tucker and Park, 2019). The signal allowing the mitochondrial targeting is a hydrophobic β-harpin motif that interacts with Tom20, another subunit of TOM complex (Jores et al., 2016). The final assembly of VDAC into the OMM is achieved by the presence of a second complex, the sorting and assembly machinery (SAM) complex (also known with the acronym of TOB complex). Again, the main SAM complex subunit is the β-barrel protein Sam50/Tob55 (Kozjak et al., 2003; Takeda et al., 2021).

The expression levels of the two yeast VDAC isoforms appears profoundly different. A recent determination of the mitochondrial proteome at high-confidence identified an average copy number of 16,000 for yVDAC1 and 1–2 copy for yVDAC2 per single mitochondria (Morgenstern et al., 2017). This difference was attributed to the promoter strength, as hypothesized immediately after *POR* genes discovery (Blachly-Dyson et al., 1997). In fact, if *POR2* sequence is cloned downstream *POR1* promoter, yVDAC2 protein levels resemble those of yVDAC1 in physiological condition (Blachly-Dyson et al., 1997).

**FIGURE 1:**
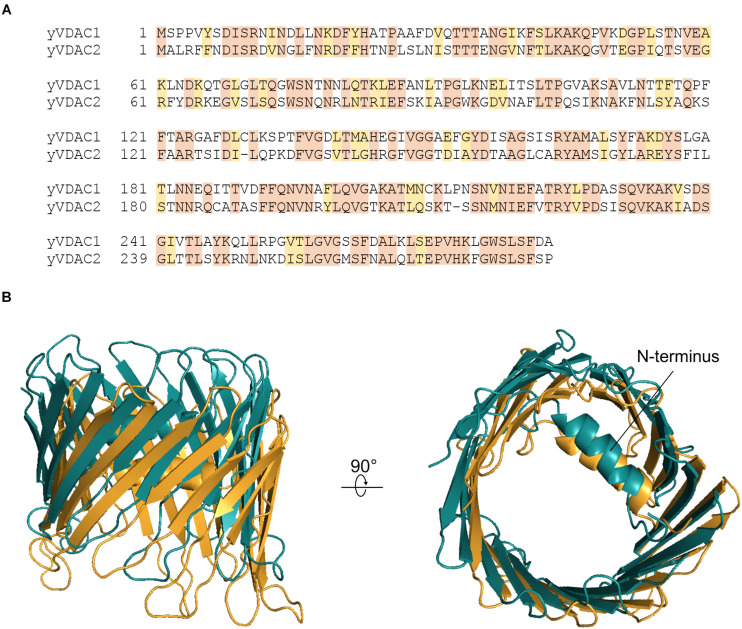
Structural features of yeast voltage-dependent anion selective channel (VDAC) isoforms. **(A)** Multi-alignment analysis of the amino acid sequences of yVDAC1 and yVDAC2 (source: GeneBank). Colors highlight the conserved (orange) or similar (yellow) residues. **(B)** Lateral and top view of the superposition of yVDACs. The predicted structure of yVDAC1 (in brigthorange) and yVDAC2 (in teal) were obtained by homology modeling using MODELLER 9.24 software and the human VDAC1 structure (PDB ID: 2JK4) as template. Final figures were drawn using PyMol software.

The primary sequences of yVDACs were determined after their identification. Multialigment analysis shown in Figure 1A revealed less than 50% of sequence identity between the two VDAC isoforms of *S. cerevisiae*. However, the computational analysis indicates high similarity in term of three-dimensional structures, as strengthened by homology modeling predictions (Guardiani et al., 2018) displayed in Figure 1B. Despite this, many substantial differences exist in the electrophysiological features, as fully described in the next paragraphs.

## Yeast VDAC1: From the Discovery to the Electrophysiological Characterization

The first evidence of porin existence in yeast was observed by Mihara et al. (1982). They described a porin-like activity in isolated OMM fractions attributed to the presence of a single predominant ∼29 kDa protein comparable to that previously found in rat liver mitochondria (Mihara et al., 1982). This protein was generically called “porin” by analogy to the other similar proteins of Gram-negative bacteria. Only after the discovery of a second porin isoform it was formally named yVDAC1 or POR1.

The primary structure of yVDAC1 was deduced from the nucleotide sequence, revealing a 283 amino acid long molecule (Mihara and Sato, 1985). In comparison to the human homologous, it has similarity and identity values of 67 and 24%, respectively (Hiller et al., 2010).

The electrophysiological properties of yVDAC1 were then investigated after protein isolation from mitochondria and incorporation into planar lipid bilayer (PLB). The protein showed a high propensity to form pores in artificial membranes, characterized by an average conductance of ∼4 nS in 1 M KCl solution (Forte et al., 1987a). In a similar manner to what previously observed for VDACs extracted from *N. crassa* (Freitag et al., 1982), rat brain (Ludwig et al., 1986), and other mammalian tissues (De Pinto et al., 1987), the application of increasing positive and negative voltages, from 0 to ±60 mV, promoted a significative reduction of yVDAC1 conductance. In particular, a high-conducting or open state was observed at low voltages. Conversely, the application of potential, starting from ±10–20 mV, resulted in a symmetrical switch toward low-conducting or closed state(s) (Forte et al., 1987a). Notably, these data were recently confirmed by our group. In particular, we observed an average value of yVDAC1 conductance of ∼4.2 nS and a voltage-dependent behavior starting from ±20–30 mV (Guardiani et al., 2018).

The ion selectivity of yVDAC1 was also investigated. The protein prefers anion over cations in the open state, while in the closed state it becomes less anionic or more cation selective (Schein et al., 1976; Forte et al., 1987b; Colombini, 2016). These observations are in agreement with our recent report showing a ratio Cl^–^:K^+^ of 2:1 in the open state and 1:4 in the closed state (Guardiani et al., 2018). Similar electrophysiological features were detected for human VDAC1 (Reina et al., 2013) and for *Drosophila melanogaster* VDAC1 (De Pinto et al., 1989b).

**FIGURE 2:**
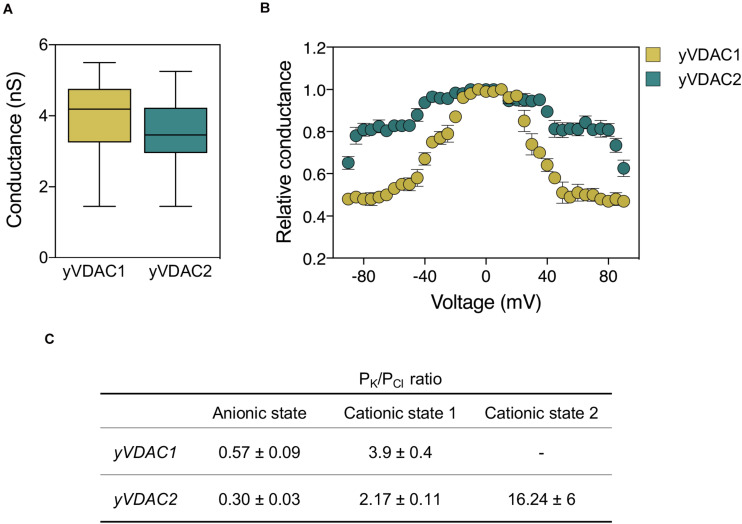
Comparative analysis of the electrophysiological features of native yVDAC1 and yVDAC2 at the Planar Lipid Bilayer. **(A)** Analysis of conductance after yVDACs reconstitution into the artificial membrane. Experiments were performed with an applied constant voltage of +10 mV in 1 M KCl solution. **(B)** Analysis of the voltage-dependence of yVDACs isoforms. Experiments were performed by gradually increasing the voltage from 0 to ±100 mV. Data are expressed as mean of the relative conductance ± SEM. The relative conductance was calculated as the *G*/*G*_0_, where *G* is the conductance at the given voltage while *G*_0_ is the conductance values calculated at 0 mV applied. **(C)** Analysis of current-voltage performed in a 10-fold gradient 1/0.1 M cis/trans KCl in a voltage ramp with amplitude ± 60 mV. Values of reversal potential were used to estimate the permeability ratio of cations (*P*_*K*_) over anions (*P*_*Cl*_) by using the Goldman-Hodgkin-Katz equation in the three states.

A summary of the main electrophysiological features, as well as a comparison with those of yVDAC2, is shown in Figure 2.

Particularly interesting for the maintenance of electrophysiological features of VDACs is the N-terminal domain, as revealed by mutagenesis experiments. E.g., the mutations of Asp 15 to Lys or Lys 19 to Glu modified the sensitivity of yVDAC1 to the voltage applied, as well as the ion selectivity (Blachly-Dyson et al., 1990; Thomas et al., 1993). Remarkably, these residues are conserved in mammalian VDACs suggesting that they are essential for the proper functioning and gating of the channel. Also truncation or substitution of specific part of the N-terminus has similar effects. E.g., the truncated yVDAC1, missing the first 8 amino acids, showed an abnormal channel activity and a pronounced instability of the open state, which rapidly switched toward multiple low-conducting states (Koppel et al., 1998).

The importance of the N-terminus for the channel function was demonstrated also for human VDAC isoforms. It is known that VDAC3 is the less active one in complementation assay of porin-less yeast performed on glycerol at 37°C or in the presence of acetic acid (a cell death inductor, Reina et al., 2010). Swapping experiments in which the first 20 N-terminal residues of VDAC3 were replaced with those from VDAC1 or VDAC2, showed increased life span and resistance to oxidative stress than porin-less yeast transformed with plasmids carrying wild-type VDAC3 sequence (Reina et al., 2010).

## Yeast VDAC1 Is Essential for the Proper Maintenance of Mitochondria

VDAC1 is by far the most abundant protein of yeast mitochondria, accounting for 7,000–19,000 copies per organelle, as for growth on glucose and glycerol, respectively (Morgenstern et al., 2017). Notably, this number overcomes of one and two orders of magnitude the copy number of the second and the third OMM most represented proteins, Tom40 and Sam50. From this study, we tried to estimate the overall OMM conductance, given by all the β-barrel proteins, and the specific contribution of yVDAC1. In this calculation, we included the putative pore-forming proteins recently discovered by Krüger et al. (2017), such as Mim1, Ary1, OMC7, and OMC8 as well as the β-barrel component of TOM and SAM complexes that can mediate small molecules exchanges across the OMM (Kmita and Budzińska, 2000; Antos et al., 2001). From this analysis emerged that the outer membrane of a single mitochondrion has an estimated permeability of ∼30 μS, 27 of which are provided by yVDAC1 (Magrì et al., 2020). It thus is clear that this isoform is mainly involved in the metabolic exchanges and in the maintenance of the communication between mitochondria and the rest of the cell.

**FIGURE 3:**
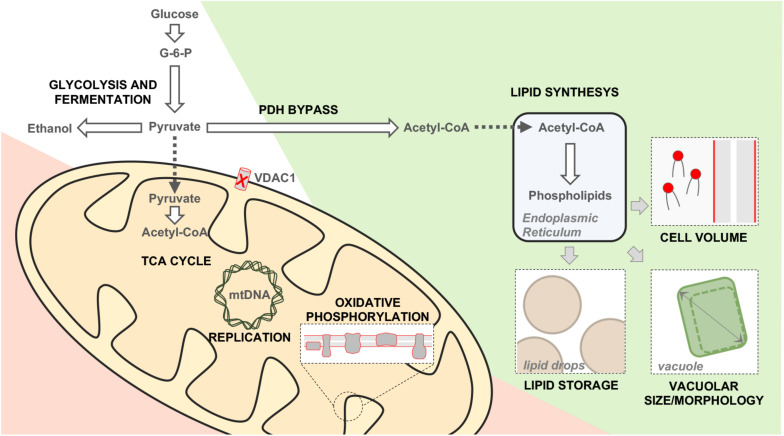
Metabolic and phenotypic changes occurring in Δ*por1* cells. Schematic representation of the main changes occurring in yeast upon *POR1* gene inactivation. Metabolic down-regulated, upregulated or unchanged pathways are displayed in red, green and white, respectively. The absence of yVDAC1 reduces the nucleotides trafficking within mitochondria, affecting the replication/expression of the mtDNA. The mitochondrial utilization of pyruvate, TCA cycle and the functioning of the electron transport system are strongly compromised. On the other hand, the cytosolic conversion of pyruvate into acetyl-coA is enhanced (PDH by-pass). The latter is addressed toward the synthesis of phospholipids, which are then stored into lipid drops (an energy reservoir). Furthermore, the newly synthetized phospholipids contribute to build plasma membrane and to increase the vacuole dimension. As a consequence, the size of Δ*por1* cells is increased of about 30% in comparison to the wild-type.

Many information about yVDAC1 function arose from the study of Δ*por1* mutant, in which *POR1* was genetically inactivated. The strain was still viable, but it showed a marked growth impairment on media containing non-fermentable carbon sources (i.e., substrates which are mainly metabolized in mitochondria) at temperature of 37°C (Blachly-Dyson et al., 1990). More recently, our group performed an extensive characterization of Δ*por1* yeast in order to expand the knowledge of this model. Our results indicate that mitochondrial respiration is dramatically compromised in the absence of yVDAC1, since the expression of the respiratory chain subunits encoded by mtDNA, but not nuclear DNA, was completely abolished, as a consequence of the dramatic reduction of mtDNA (Magrì et al., 2020). In this context, the metabolites commonly addressed to the mitochondria, as pyruvate, are pushed toward a cytosolic utilization and the whole cell metabolism undergoes a complete rearrangement aimed to by-pass mitochondria. To survive in the absence of yVDAC1, the cells enhance the biosynthesis of phospholipids, which are then stored into lipid droplets (as an energy reserve) or in the plasma membrane, as schematized in Figure 3 (Magrì et al., 2020). Overall, these results revealed once again the irreplaceable role of yVDAC1 for the OMM permeability and for mitochondrial metabolism.

An increasing body of evidence suggests an equally important function of yVDAC1 in mitochondrial biogenesis. It is known since many years that the inactivation of *POR1* affects the expression of specific subunits of TOM and SAM complexes (Galganska et al., 2008; Karachitos et al., 2009). However, only recently a direct involvement of yVDAC1 in this mechanism was demonstrated. The assembly of TOM complexes requires the presence of the constituent protein Tom40 and Tom22 (Model et al., 2001). Sakaue et al. (2019) demonstrated that yVDAC1 interacts with Tom22, preventing the transition from a dimeric to a trimeric form of the complex, essential for the import of specific proteins. Furthermore, yVDAC1 antagonizes Tom6, another regulator of TOM assembly (Sakaue et al., 2019). At the same time, yVDAC1 modulates also the Translocase of the inner membrane (TIM) complex activity, by its direct interaction with Tim22. In this contest, yVDAC1 was individuated as a coupling factor for protein translocation of carrier precursors into the inner mitochondrial membrane (IMM) (Ellenrieder et al., 2019). Notably, both these works supported this brand new role of yVDAC1 independent of its metabolic function (Edwards and Tokatlidis, 2019).

As a last point, the role of yVDAC1 in the regulation of yeast redox homeostasis is less characterized than in mammals. VDAC are intrinsically sensitive to oxidation (Saletti et al., 2017, 2018) and during the exponential and/or stationary growth phases they undergo oxidation/carbonylation (O’Brien et al., 2004). However, this effect is exacerbated in yeast strains devoid of the antioxidant enzymes superoxide dismutase (SOD), as expected. The cytosolic Cu/Zn SOD (SOD1), however, not only protects yVDAC1 from oxidation but it is required for the proper channel activity and expression of yVDAC1 (Karachitos et al., 2009). At the same time, the inactivation of *POR1* affects the expression of the mitochondrial Mn-SOD (SOD2) (Galganska et al., 2008, 2010), suggesting a mutual regulation between the two proteins.

## The Controversial Story of yVDAC2

Until 1996, yVDAC1 was believed the only porin isoform of *S. cerevisiae*. Two different events contributed to the discovery of the second VDAC: the availability of yeast genomic sequences and the increasing use of recombinant techniques aimed to inactivate specific genes. By the insertion of a functional *LEU2* gene in the *POR1* sequence, yVDAC1 was knocked-out and the Δ*por1* mutant was obtained (Dihanich et al., 1987). The mutant showed normal levels of other OMM proteins but reduced levels of cytochrome c_1_ and cytochrome oxidase subunit IV (Dihanich et al., 1987). Surprisingly, Δ*por1* strain was still viable, even if it grew slower than the wild-type at 30°C (Dihanich et al., 1987). This result suggested the existence of some unknown alternative pathway through which small metabolites and ions could cross the OMM.

The analysis of Δ*por1* growth on glycerol at the elevated temperature of 37 °C has revealed a specific defect (Blachly-Dyson et al., 1990). Glycerol, indeed, is a non-fermentable carbon source that forces the utilization of mitochondria (Gancedo et al., 1968). By screening a genomic *S. cerevisiae* library, Blachly-Dyson et al. (1997) identified a second VDAC isoform through its ability to correct this Δ*por1* growth defect. Then, a second porin gene, called *POR2* and encoding for yVDAC2, was individuated.

yVDAC2 was immediately indicated as a potential yVDAC1 substitute, even if some peculiarities emerged. For instance, the second yeast VDAC isoform was able to restore the growth defect of Δ*por1* only upon specific conditions. If *POR2* is present in low copy number (one or two copies per cell) it fails to substitute yVDAC1. Conversely, when *POR2* sequence is cloned downstream the *POR1* promoter it can successfully restore the yeast growth as in wild-type (Blachly-Dyson et al., 1997). Notably, Δ*por1* transformation with single copy plasmid, carrying the encoding sequences of mouse VDAC isoforms 1 and 2, completely recovers the yeast growth on glycerol at 37°C (Sampson et al., 1997). This suggested that yVDAC2 had pore-forming activity but such compensation was strictly depended on its concentration. However, all the efforts made by Blachly-Dyson et al. (1997) to isolate and incorporate yVDAC2 in artificial membrane failed, prompting the scientific community to question the pore-forming ability of yVDAC2 and its involvement in mitochondrial bioenergetics. This idea was supported by the low level of similarity between VDAC isoforms, consisting in only 49% (see Figure 1A). Also, not many information was obtained from deletion studies: differently from Δ*por1*, the genetic inactivation of *POR2* gene does not affect yeast growth in any condition, while the simultaneous absence of both endogenous porins, as in the double mutant Δ*por1*Δ*por2*, only exacerbates the yeast growth defect on glycerol typical of Δ*por1*.

## Yeast VDAC2 Has Pore Forming Activity and a Peculiar Ion Selectivity

After its discovery, the interest in yVDAC2 has waned for almost two decades. However, in 2016, investigating the role of human SOD1 in yeast, we casually noticed a complete recovery of Δ*por1* growth defect on glycerol at 37°C in the presence of overexpressed hSOD1. In this condition, the expression level of *POR2*, as well as of other genes encoding for OMM β-barrel proteins (Tom40 and Sam50), was found significantly increased (Magrì et al., 2016b). Since the same results did not appear in Δ*por1*Δ*por2* yeast, we hypothesized that yVDAC2 might re-establish the OMM pore activity.

To definitely clarify this aspect, we established a collaboration with the group of prof. Hanna Kmita (Poznan, PL), aimed at purify with high yield yVDAC2. In the first attempt Δ*por1* strain was transfected with a plasmid carrying *POR2* sequence and the protein was purified from Δ*por1* mitochondria, avoiding yVDAC1 contamination (Guardiani et al., 2018). It was called *native* yVDAC2. The second strategy applied the heterologous expression of a 6xHis-tagged yVDAC2 in *E. coli* (Magrì et al., 2019). Being a membrane protein, yVDAC2 localized in the inclusion bodies and needed high concentrations of denaturant to be purified, followed by a refolding step in presence of specific detergents (Engelhardt et al., 2007). Remarkably, this refolding method was successfully applied many times and by different groups, producing VDAC proteins with indistinguishable features from those native (Ujwal et al., 2008, Checchetto et al., 2014; Okazaki et al., 2015; Magrì et al., 2016a; Reina et al., 2016a; Queralt-Martín et al., 2020).

The electrophysiology at the PLB revealed that both native and recombinant proteins were able to form pores with the same, typical VDAC-like conductance of ∼3.6 nS in 1 M KCl, as displayed in Figure 2A) (Guardiani et al., 2018; Magrì et al., 2019). As for voltage-dependence, native yVDAC2 resembled yeast or human VDAC1, even if it began to close at ±40–50 mV. This suggests that yVDAC2 is slightly less sensitive to the applied voltage (Figure 2B). However, this specific aspect was amplified in the recombinant protein, which began to close only at ± 80–90 mV (Magrì et al., 2019). The difference between the native and recombinant forms of yVDAC2 raises the interesting question of whether the native yVDAC2 was subject to specific PTMs, not occurring in the heterologous expression in *E. coli*. The influence of PTMs in VDAC activity is indeed a rather unexplored subject.

Ion selectivity of yVDAC2 was particularly interesting. The computational analysis revealed a similar tridimensional structure for the two yeast VDAC isoforms but a net charge of +11 in the case of yVDAC2, in comparison to +1 of yVDAC1. Thus, anion selectivity was expected for yVDAC2 in the open state, as also predicted by bioinformatic analysis, with a chloride selectivity estimated 2–3 times higher than that displayed by isoform 1 (Guardiani et al., 2018). The analysis of native yVDAC2 at the PLB allowed the identification of up three states with different parameters of ionic selectivity: two of them appeared to be high-conductance states but with opposite selectivity (Guardiani et al., 2018; Magrì et al., 2019). In the open state, the ratio Cl^–^: K^+^ for yVDAC2 was 3:1, definitely more anionic than the corresponding state displayed by yVDAC1. The second high-conducting state showed a prominent cation selectivity (Cl^–^: K^+^ = 1:2) (Guardiani et al., 2018). This oddity is not entirely new with VDACs: a similar state was already observed for VDAC1 from mammals (Pavlov et al., 2005). The third state detected by studying yVDAC2 ion selectivity was a low-conducting and very cation-selective state (Cl^–^: K^+^ = 1:16, Guardiani et al., 2018). Such state was previously unseen in any studied VDAC. The ion selectivity of yVDAC isoforms is detailed in Figure 2C.

## What Is yVDAC2 Function?

Despite its demonstrated pore-forming activity, all evidences suggest that yVDAC2 plays only a marginal role in mitochondria bioenergetics. Indeed, the deletion of *POR2* does not significantly affect yeast growth on glycerol at 37°C although its simultaneous inactivation with *POR1* aggravates the growth defect (Blachly-Dyson et al., 1997). The involvement of yVDAC2 in mediating the OMM permeability to small molecules, such as NADH, was studied in Δ*por1* cells. Here, NADH permeability was found 20 times lower than in wild-type (Lee et al., 1998). However, similar results were obtained for the double mutant Δ*por1*Δ*por2*, excluding definitely the involvement of yVDAC2 in this pathway. Later, Tom40 was indicated as a valid substitute of yVDAC1 in Δ*por1* cells (Kmita and Budzińska, 2000; Antos et al., 2001).

A participation in the maintenance of energy homeostasis was also proposed for yVDAC2. SNF1 protein kinase is the orthologue of the mammalian AMP-activated protein kinase (AMPK), both important players in the regulation of cell growth and glucose metabolism in response to the energy limitation (Hedbacker and Carlson, 2008; Mihaylova and Shaw, 2011). It was shown that SNF1 co-precipitated with both yeast VDACs and SNF1 function was significantly affected only when both porin genes are simultaneously inactivated (Strogolova et al., 2012). For this reason, yVDAC2 was identified as a “co-sensor” of a stress signal upstream of SNF1, even if the precise mechanism was still unclear.

Anyway, given the pacucity of literature information, the residual hypothesis is that yVDAC2 acts as a rescue permeability mitochondrial pathway, expressed in presence of some undefined stimulus. In fact, the absence of yVDAC1 *per se* is not able to activate *POR2* gene expression (Magrì et al., 2020). On the contrary, the co-presence of an additional factor, such as the overexpressed hSOD1, induces *POR2* expression and restores the yeast growth defect of Δ*por1* cells (Magrì et al., 2016b).

## Δ*Por1* Yeast, an Opportunity to Study VDAC Role in Human Pathologies

Despite the obvious absence of a nervous system in yeast, basic mechanisms and pathways underlying neurodegenerative diseases, such as mitochondrial dysfunction, transcriptional dysregulation, trafficking defects and proteasomal dysfunction, are extremely well conserved between humans and yeast, enabling detailed studies of the molecular events involved in those conditions.

Mitochondrial dysfunctions, along with defects in proteasomal activity and misfolded protein aggregations, are well-known molecular hallmarks of neurodegenerative disorders that can be easily recapitulated in a relatively simple system as the yeast. This is made possible by the presence of disease-associated human orthologues or by the introduction of a human protein directly linked to the disease of interested with easy manipulation techniques. For instance, yeast has been successfully used to investigate TDP43 and FUS dysfunction in amyotrophic lateral sclerosis (ALS), amyloid-β peptide and Tau in Alzheimer’s disease, α-synuclein (αSyn) and Lrrk2 in Parkinson’s disease, and Huntingtin in Huntington’s disease (as reviewed in Miller-Fleming et al., 2008; Bharadwaj et al., 2010; Pereira et al., 2012; Rencus-Lazar et al., 2019). In this contest, VDAC proteins (and VDAC1 in particular) play a crucial role in mediating mitochondrial dysfunction. In fact, most of the previously cited proteins are able to aggregate onto the cytosolic surface of mitochondria using VDAC as an anchor point (Magrì and Messina, 2017). Thus, the use of Δ*por1* mutant, transformed or not with plasmids carrying encoding sequences for human VDAC isoforms or mutants, represents an important opportunity to clarify the specific roles of porins in pathological contexts.

The involvement of human VDAC1 in mediating αSyn toxicity in Parkinson’s disease was demonstrated for the first time in yeast. Rostovtseva et al. (2015) introduced αSyn expression in the Δ*por1* mutant, noticing a yeast growth defect on galactose only when the protein was expressed together with the human VDAC1. This finding supports the idea that mitochondrial dysfunction mediated by αSyn occurs through the modulation of VDAC1 permeability (Rostovtseva et al., 2015). Also, the specific ability of the three VDAC isoforms to counteract oxidative stress was investigated in yeast (Galganska et al., 2008), as well as the antibiotic minocycline specificity to interact with VDACs. These last studies revealed that minocycline interacts in a different manner with VDAC proteins and only isoform 3 is able to mediate the cytoprotective effect counteracting H_2_O_2_-mediated toxicity (Karachitos et al., 2012, 2016).

In the light of the emerging consideration of VDAC proteins as a pharmacological target in many diseases (Magrì et al., 2018; Shoshan-Barmatz et al., 2020), these few examples highlight the potential usage and the versatility of Δ*por1* cells for biotechnological and biomedical application.

## Conclusion

Along with the increased interest of the scientific community in understanding VDACs role in apoptosis and mitochondrial dysfunctions, many studies have been carried out on mammalian or human porins, but significantly fewer for the yeast counterparts. Nevertheless, the complete understanding of *S. cerevisiae* VDACs functioning is equally important, especially considering its potential use as biomedical/biotechnological tool. The aim of this review was to collect all the information present in the literature about both yeast VDAC isoforms and to depict a framework as complete as possible. Despite this, several questions need to be addressed yet and deserved to be answered. One of above all: what is the physiological role of yVDAC2? Given the peculiar electrophysiological features here listed, it is indeed hard to believe that this protein is only a genetic heritage from a duplication event.

## Author Contributions

MCDR, FG, and SCN collected the information and prepared the reference list for the manuscript. AM drow the figures and wrote the manuscript. VDP supervised the work. All authors have read and approved the manuscript.

## Conflict of Interest

FG, AM, and VDP are affiliated with we. MitoBiotech S.R.L, a spin-off company to the University of Catania. All the authors declare that the research was conducted in the absence of any commercial of financial relationships that could be construed a potential conflict of interest.
